# M. Guepin on Punctures of the Eye

**Published:** 1844-02-24

**Authors:** 


### M. GUEPIN ON PUNCTURES OF THE EYE.

M. D. an architect, brought his son to M. Guépin, in 1843. A minute fragment of iron had entered his eye, reaching from the cornea to the capsule of the crystalline lens, and almost touching the iris. It was impossible to grasp the fragment, and an incision would have been difficult, as it lay upon the upper edge of the pupil. M. Guepin accordingly devised the following remedy. He prescribed a collyrium made with distilled water and acetic acid, being persuaded that if the fragment became oxidized at its corneal extremity, the oxidation would spread over its whole surface, and that the dissolution and absorption of the fragment would follow. The event justified his supposition. At the end of three weeks, the cure was complete, with the exception of an almost imperceptible white point upon the capsule, and a very slight cicatrix on the cornea. In another case, the same collyrium was again used with success to carry off the oxide of iron left in the substance of the cornea by a fragment of iron which had remained in it a considerable time.—*Lond. Med. Gaz. from Ann. d'Oculistique.*

